# The Role of Aquaporin 4 in Lacrimal Gland Ductal Fluid Secretion in Mice

**DOI:** 10.1167/iovs.65.5.30

**Published:** 2024-05-21

**Authors:** Gréta Elekes, Virág Csapó, Dóra Szarka, László Szalay, Marietta Margaréta Korsós, Dorottya Tálosi, Dénes Török, Edit Tóth-Molnár

**Affiliations:** 1 Department of Ophthalmology, University of Szeged, Szeged, Hungary; 2 Department of Pharmacology and Pharmacotherapy, University of Szeged, Szeged, Hungary; 3 Albert Szent-Gyorgyi Medical School, University of Szeged, Szeged, Hungary; 4 Department of Anatomy, University of Szeged, Szeged, Hungary; 5 Faculty of Health Sciences and Social Studies, University of Szeged, Szeged, Hungary

**Keywords:** aquaporin 4 (AQP4), lacrimal gland (LG), duct epithelium, mouse

## Abstract

**Purpose:**

Earlier reports highlighted the predominant presence of aquaporin 4 (AQP4) in the duct cells of rabbit lacrimal glands (LGs). Whereas significant alterations in AQP4 mRNA levels have been observed in experimental dry eye and during pregnancy, the impact of AQP4 in LG ductal fluid production remains unclear. In our recent work, the role of AQP4 in LG ductal fluid secretion was investigated utilizing wild type (WT) and AQP4 knock out (KO) mice.

**Methods:**

Tear production was assessed in both WT and KO animals. Immunostaining was used to identify AQP4 protein. Duct segments were harvested from LGs of WT and KO mice. Fluid secretion and filtration permeability (P_f_) were quantified using video-microscopy. Ductal tear production, elicited by a cell-permeable cAMP analogue (8-bromo cAMP), carbachol, vasoactive intestinal peptide (VIP), and phenylephrine (PHE), were assessed in both WT and KO ducts.

**Results:**

A higher expression of AQP4 protein was noted in the duct cells from WT mice when compared to acinar cells. P_f_ did not show notable alterations between WT and AQP4 KO ducts. Carbachol elicited comparable secretory responses in ducts from both WT and KO animals. However, 8-bromo cAMP, VIP, and PHE stimulation resulted in decreased secretion in ducts from AQP4 KO LGs.

**Conclusions:**

Our findings underscore the functional relevance of AQP4 in the fluid production of mouse LG ducts. AQP4 seems to play different roles in fluid secretions elicited by different secretagogues. Specifically, cAMP-mediated, and adrenergic agonist-related secretions were reduced in AQP4 KO ducts.

The lacrimal gland (LG) is the major source of tear fluid. Whereas acinar cells produce the higher part of fluid volume, molecular-genetic and immunofluorescent transmembrane transporter patterns of duct cells have long suggested that ducts may play a determining role in ion and fluid secretion in addition to their drainage function.^1^^–^^5^

Introduction of new experimental methods, such as duct isolation technique and video-microscopy, has allowed functional investigation of isolated duct segments.^6^^,^^7^ Using these methods, the fluid secretory capability of LG ducts from different species has been demonstrated and the involvement of several transmembrane transporters in the tear secretion of the ducts have been reported.^8^^,^^9^ Autonomic regulation of ductal function has also been investigated.^10^ These accumulating experimental results provide compelling evidence that ductal cells possess an essential, albeit species-dependent function in the formation of the final secreta of the LG.^11^

Lacrimal duct fluid production is an osmotic process. The combined actions of basolateral and apical ion channels create an osmotic gradient, which determines the vectorial movement of water. Although the ability of LG ducts to secrete fluid has been demonstrated, the route of transepithelial water movement is not yet fully understood. Whereas paracellular water flow is the dominant component of fluid transport, transmembrane water channels, known as aquaporins (AQPs), possess a key role in various epithelial cells. AQPs constitute a group of transmembrane protein structures that enable bidirectional transport of water and small uncharged molecules through the cell membrane. The regulatory mechanisms of AQPs in the secretory activity of various exocrine glands is extensively researched.^12^^–^^15^ The presence of several AQPs (AQP1, 2, 3, 4, 5, 7, 8, 9, and 11) has been confirmed in the LG so far, however, their role in LG secretion remains controversial. In gene expression studies and immunofluorescence experiments, the predominant presence of AQP4 is found in the ducts, whereas AQP5 was abundant in the acinar cells both in the rabbit and mouse.^16^^–^^19^

In an earlier report by Moore et al., both basal and stimulated tear production in AQP1, AQP3, AQP4, or AQP5 knock out (KO) mice proved to be unchanged providing direct evidence against the essential involvement of these AQPs in LG fluid secretion.^20^ Sasaki et al. reported similar results of unaffected tear secretion in AQP5 KO mice.^21^ However, in a recent report, decreased tear secretion was demonstrated in AQP5 KO mice.^22^ Defective cellular trafficking and altered distribution of the AQP5 protein were demonstrated in a mouse model of Sjögren syndrome, providing evidence of AQP5 involvement in LG function.^23^^,^^24^ On the other hand, significant alterations of AQP4 and 5 were reported in 2 rabbit models of dry eye (rabbits with experimental autoimmune dacryoadenitis and pregnant rabbits). Expression patterns of AQP4 in the LGs from gravid rabbits demonstrated substantial alterations at both gene and protein levels in acinar and ductal cells. AQP4 mRNA levels were reduced in the duct cells derived from pregnant animals, aligning with results from LG ducts of rabbits with experimental autoimmune dacryoadenitis. These significant alterations of AQP4 in LG ducts lend support to the theory that AQP4 may actively participate in the fluid secretion of the LG.^18^^,^^19^ However, there is a growing body of data regarding the role of AQPs in LG secretion, yet these reports remain controversial in various aspects.

The availability of transgenic mouse models lacking different AQP water channel holds great promise in clarifying the function of AQPs in ductal tear production. In the present study, isolated duct segments from wild type (WT) and AQP4 KO mice LGs were used to investigate the effect of various secretory agents in the presence and absence of the AQP4 channel. Osmotic water permeability (filtration permeability [P_f_]) of WT and AQP4 KO duct epithelia was also calculated.

## Materials and Methods

### Animals

AQP4 transgenic mouse strain developed by Dr. Alan Verkman (University of California, Irvine, CA, USA) was used in our investigations. To determine the genetic background of the animals, genomic DNA samples were prepared from the tail of the mice to perform traditional PCR (Phire Tissue Direct PCR Master Mix; Thermo Fisher Scientific, Vilnius, Lithuania). The mice were housed at a temperature of 23°C to 25°C, under a 12-hour light-dark cycle, and provided ad libitum access to standard chow and water. The WT refers to the +/+ littermates of the AQP4 KO mice. The age of the animals were 14 to 18 weeks, the sex ratio was 1:1.

Mouse LGs of WT and AQP4 KO mice were used throughout the study. Intraperitoneal ketamine (80 mg/kg) and xylazine (10 mg/kg) injections were used for narcosis and pentobarbital overdose (100 mg/kg) was administered to euthanize the animals.

All experiments were carried out in accordance with the ARVO Statement for the Use of Animals in Ophthalmic and Vision Research. The protocol was accepted by the Ethical Committee for the Protection of Animals in Research of the University of Szeged, Szeged, Hungary. The protocol adhered to the Directive 2010/63/EU of the European Parliament.

### Solutions and Chemicals

Media and its supplements for LG duct isolation and culture (Dulbecco's modified eagle medium [DMEM], McCoy's 5A tissue culture medium, fetal bovine serum [FBS], L-glutamine, bovine serum albumin [BSA]), carbachol (carbamoylcholine chloride), 8-bromoadenosine 3′5′-cyclic monophosphate (8-bromo cAMP), vasoactive intestinal peptide (VIP), and phenylephrine (PHE) were ordered from Sigma-Aldrich Corp. (Budapest, Hungary). Purified collagenase was acquired from Worthington Biochemical Corp. (Lakewood, NJ, USA). For immunostaining experiments, VPAC1 and VPAC2 primary rabbit polyclonal antibodies and Alexa Fluor 488 secondary goat anti-rabbit antibody was obtained from Abcam (Cambridge, UK), AQP4 primary rabbit polyclonal antibody derived from Alomone Labs (Jerusalem, Israel).

The composition of solutions used in our experiments is summarized in the Table. The pH of standard HEPES-buffered solution was set to 7.4 with HCl at 37°C.

**Table.** Composition of Solutions Used in the Experiments

Compound	HEPES Buffered Solution	Isolation Solution	Storage Solution	Culture Solution
NaCl, mM	140	–	–	–
KCl, mM	5	–	–	–
Na-HEPES, mM	10	–	–	–
MgCl_2_, mM	1	–	–	–
CaCl_2_, mM	2	–	–	–
D-Glucose, mM	10	–	–	–
Dulbecco modified eagle medium	–	X	X	–
Collagenase, U/mL	–	100	–	–
Bovine serum albumin, mg/mL	–	1	0,03	–
McCoy's 5A tissue culture medium	–	–	–	X
Fetal bovine serum, vol/vol %	–	–	–	10
L-glutamine, mM	–	–	–	2

### Measurement of Tear Secretion

Tear secretion was measured in anesthetized mice by applying phenol red impregnated cotton threads (Zone-Quick, Showa Yakuhin Kako Ltd., Japan) to the lateral canthus of both eyes for 5 minutes. The threads turned red upon contact with tears, and the wetting length was expressed in millimeters.

### Immunofluorescence

Immunofluorescence was used to detect and localize AQP4 protein in the LGs.

Immunofluorescence staining was used to examine and compare the density of VIP receptors (VPAC1 and VPAC2) in the two types of ducts with the aim of elucidating whether the absence of AQP4 channels influences the expression pattern and the abundance of VIP receptors.

A detailed description of the protocol was provided previously by our research group.^9^ Slides were observed using a Zeiss LSM 880 confocal laser scanning microscope (Oberkochen, Germany).

### Isolation of Ducts From Mouse LGs

Interlobular and intralobular duct segments were isolated as described previously.^6^ The viable duct segments were maintained for 8 to 10 hours in a 37°C incubator gassed with 95% O_2_/5% CO_2_.

### Measurement of Ductal Fluid Secretion

A video-microscopic method was used to quantify ductal tear secretion, as previously reported.^7^ Briefly, the ends of isolated ducts seal after overnight incubation. The secretory processes resulted in an increase in the luminal volume (LV) of the ducts as the closed luminal space filled with the secreted fluid. Ductal volume changes were monitored by video-microscopy, with bright-field images captured at 1-minute intervals. The images were evaluated using Fiji (ImageJ) imaging software.^25^

### Filtration Permeability 

To analyze the water permeability of WT and AQP4 KO LG duct epithelia, osmotic permeability was calculated as previously described.^7^ The osmotic water permeability constant ([P_f_] = µm/s) was calculated from the initial volume (V_0_ = πR_0_^2^l_0_), the initial slope of the relative volume increase (d(V/V_0_)/dt), the initial luminal surface area (2πR_0_l_0_), and the molar volume of water (V_w_ = 18 × 10^12^ µm/mol):

$$\begin{array}{c} P_{f}=\left[V_{0}d\left(V/V_{0}\right)/\mathrm{dt}\right]/\left[S_{0}V_{w}\left(\mathrm{os}m_{\mathrm{in}}-\mathrm{os}m_{\mathrm{out}}\right)\right] \end{array}$$

where osm_in_ and -osm_out_ is the difference between inner and outer mediums osmolarity (osm_in_ = 290 mOsm and osm_out_ = 145 mOsm).

### Statistical Analysis

A mixed ANOVA model was used to calculate ductal fluid secretion as previously described.^9^ The data were analyzed using SigmaPlot statistical software (Systat Software Inc., London, UK) and presented as means ± SEM. A significance level of *P* < 0.05 was applied.

## Results

### Tear Secretion of WT and AQP4 KO Mice

**Figure 1.**
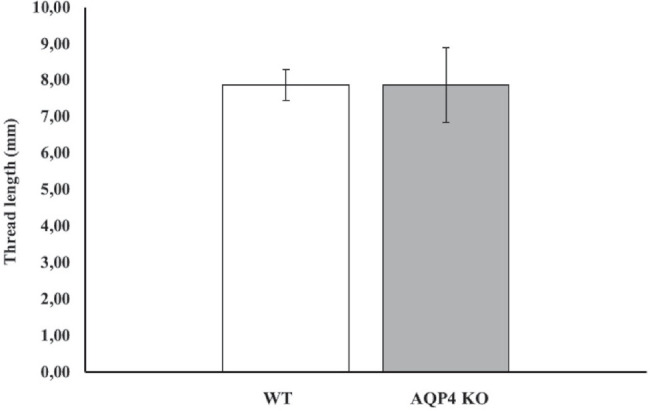
Tear secretion of WT and AQP4 KO mice. Data from both eyes of four mice (2 males and 2 females) were averaged and are presented as means ± SEM. Tear secretion of WT and AQP4 KO animals did not differ significantly.

Tear secretion was quantified in both WT and AQP4 KO mice, and measurements from both eyes were averaged. The mean amount was 7.88 ± 0.85 mm/5 minutes (*n* = 4) in the WT group, and 7.88 ± 2.06 mm/5 minutes (*n* = 4) in the AQP4 KO group (Fig. 1). The amount of tear secreted by WT and AQP4 KO animals did not vary significantly ( *P* = 0.46).

### Immunofluorescence

**Figure 2.**
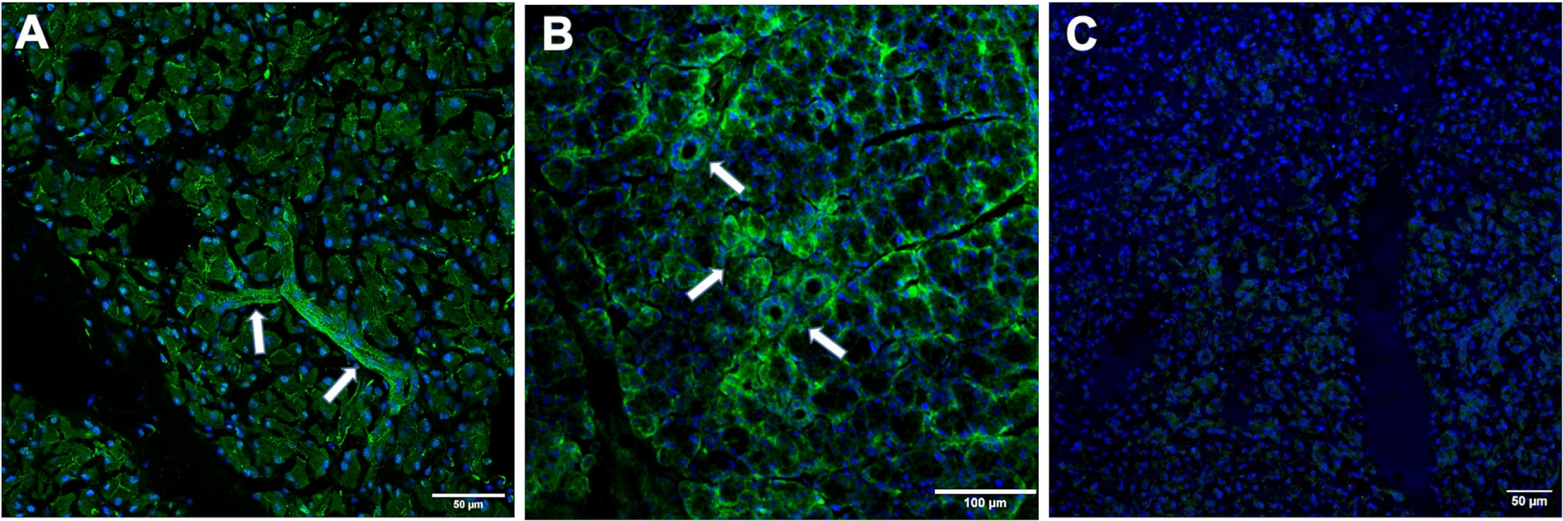
AQP4 immunofluorescence staining of LG from WT and AQP4 KO mice. (**A, B**) AQP4 staining was most prominent in the ducts. Robust AQP4 staining could be observed in the basolateral membrane of duct cells (*arrows*), and to a lesser extent in the apical side of the ducts and in the cytoplasmic area. (**C**) No AQP4 staining could be detected in LG tissues from AQP4 KO animals.

The basolateral membrane of duct cells showed robust AQP4 staining, with a lesser extent on the apical side of the ducts and within the cytoplasmic area. Additionally, punctate staining was visible in the cytoplasmic area of acinar cells. Weaker staining was detected on the basolateral side of the acinar cells (Figs. 2A, 2B). As anticipated, AQP4 protein was not evident in LG tissue samples from AQP4 KO mice (Fig. 2C).

**Figure 3.**
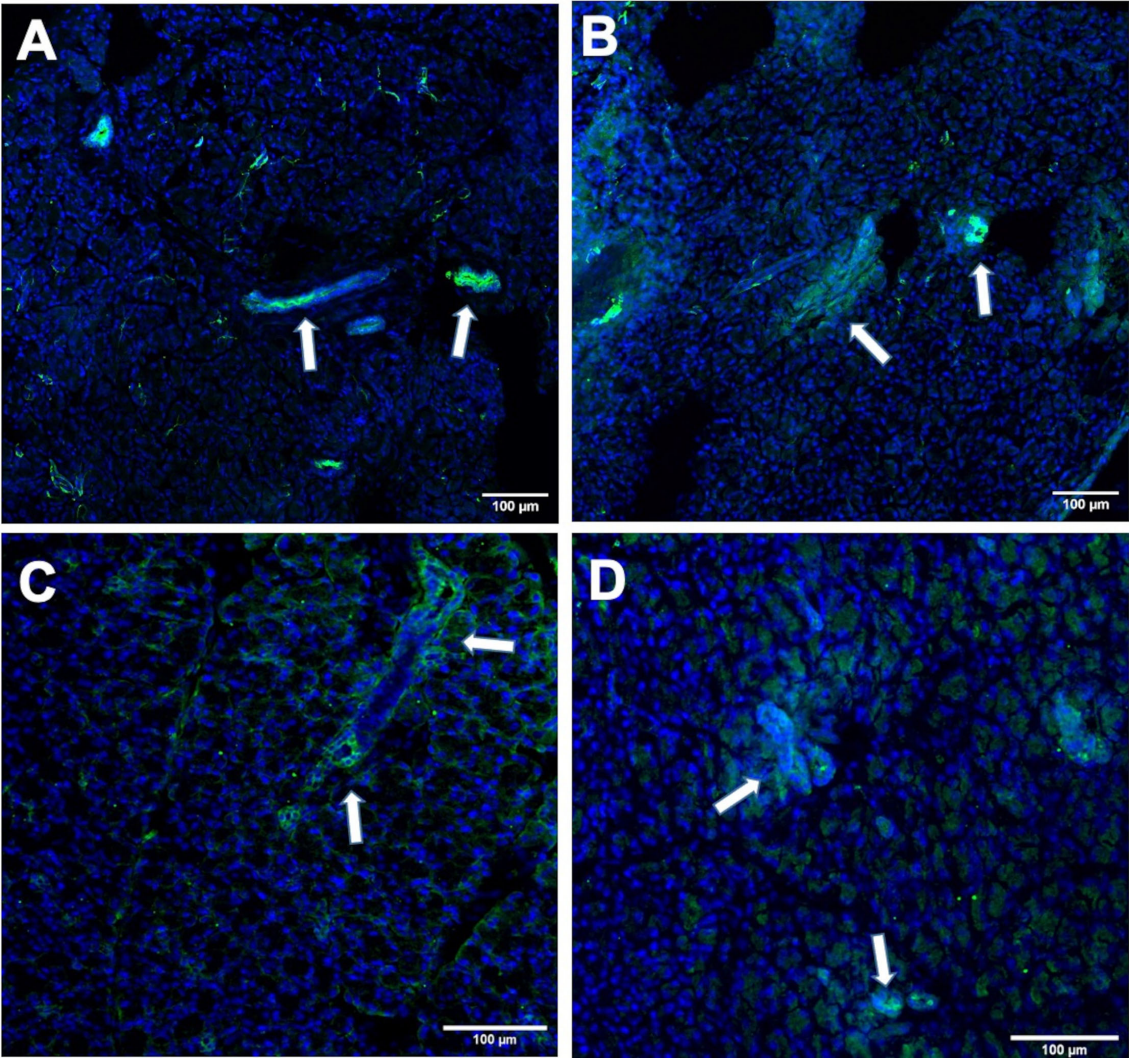
VPAC1 (panels **A** and **B**) and VPAC2 (panels **C** and **D**) immunofluorescence staining of LG from WT and AQP4 KO mice. Panel (**A**) VPAC1 staining in WT LG, panel (**B**) VPAC1 in KO LG. Panel (**C**) VPAC2 in WT LG, panel (**D**) VPAC2 in KO LG. The staining pattern proved to be similar in both WT and KO mouse LGs.

The staining pattern and density of VPAC1 and VPAC2 receptors were found to be similar between WT and AQP4 KO ducts (Figs. 3A–D). A mosaic pattern of staining was present in the LG of both WT and AQP4 KO animals. Immunoreactivity varied widely across different duct segments, ranging from no staining to strong staining.

### P_f_ of Interlobular and Intralobar LG Duct Epithelium

**Figure 4.**
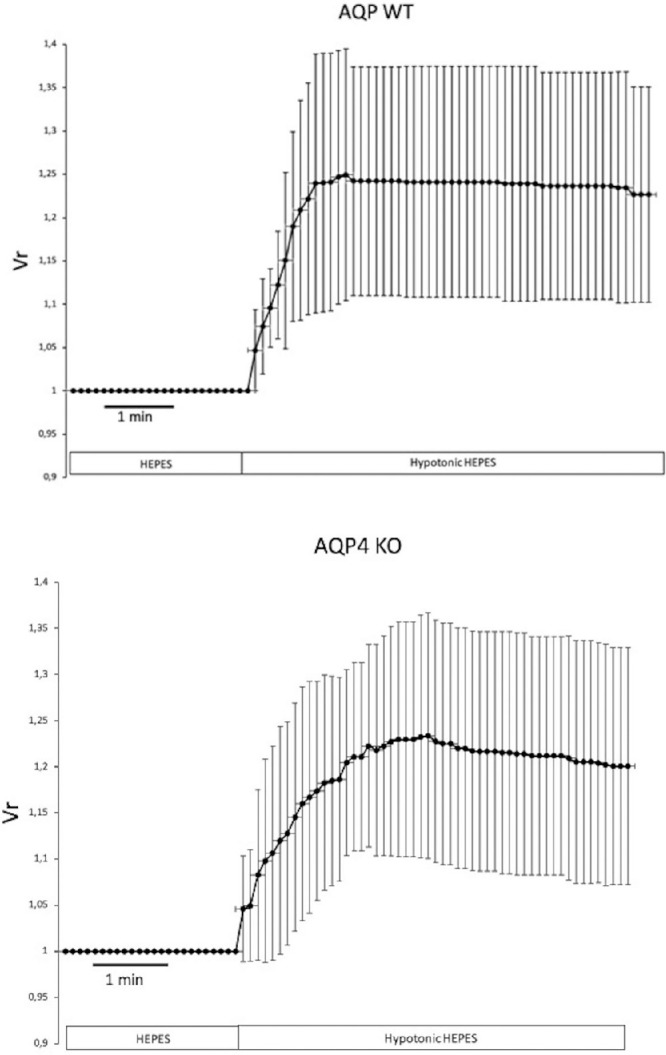
Osmotic (filtration) permeability of isolated LG ducts from WT and AQP4 KO mice. Interlobular and intralobar ducts were isolated from the LGs of WT and AQP4 KO mice. Changes in the relative luminal volume (V_r_) induced by a 50% reduction in the osmolarity of the perfusate are shown. Measurements were performed by video-microscopy, capturing images at 5 second intervals. Data were obtained from six ducts isolated from three different animals and are presented as means ± SEM. No statistically significant difference could be detected in the filtration permeability between WT or AQP4 KO ducts.

In our previous experiments, a hypotonic solution resulted in a marked swelling response in isolated rabbit LG ducts, indicating rapid water influx into the luminal space caused by the NaCl gradient.^7^ To address the role of AQP4 in the water permeability of ductal epithelium, we compared the swelling response of WT and AQP4 KO mouse ducts during hypotonic stress. Changes in the LV following the application of a hypotonic solution in the tissue bath are shown in Figure 4. No significant difference (*P* = 0.36) was observed in the hypotonic solution-induced swelling response between WT (43.7 ± 2.8 µm/s) and AQP4 KO mouse ducts (46.5 ± 3.6 µm/s, *P* = 0.36).

### Role of AQP4 in Ductal Fluid Secretion Induced by Different Secretagogues

To gain knowledge of the role of AQP4, the effects of different secretagogues on ductal fluid secretion were examined. The effects of the tested agents were measured and compared in both WT and AQP4 KO mouse ducts.

### Carbachol and VIP

**Figure 5.**
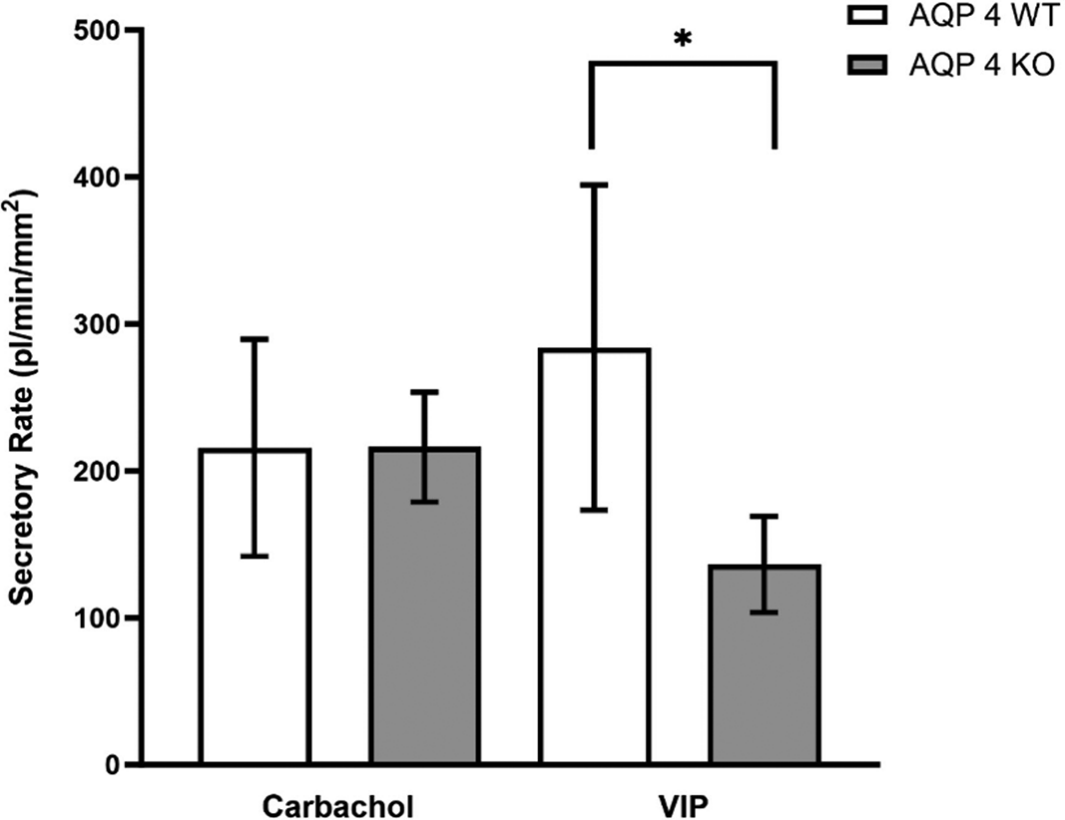
Effect of carbachol and VIP on fluid secretion in mouse ducts isolated from WT and AQP4 KO mouse LGs. WT and AQP4 KO mouse ducts were exposed to either 100 µM carbachol or 200 nM VIP. Secretory rates are shown. In both cases (carbachol and VIP) data were obtained from six ducts isolated from four different animals and are presented as means ± SEM. Carbachol stimulation was not affected by the absences of AQP4 channels, whereas the effect of VIP stimulation was significantly lower in AQP4 KO mouse ducts.

The function of different exocrine glands is mainly regulated by parasympathetic effects. To study the involvement of AQP4 in the secretory response to parasympathetic stimuli, isolated ducts were treated with carbachol or VIP in these sets of experiments. Carbachol treatment (100 µM) resulted in a notable fluid secretion to the same extent in both WT and AQP4 KO mouse ducts (WT = 215 ± 74 pl/min/mm^2^ and AQP4 KO = 216 ± 37 pl/min/mm^2^; respectively). Lack of AQP4 did not influence the secretory response to carbachol (*P* = 0.989; Fig. 5). In contrast, a remarkable difference was detected in the VIP-evoked (200 nM) fluid secretion between AQP4 KO and WT mouse ducts. Although VIP induced a definite secretory response in AQP4 KO mouse ducts (136 ± 33 pl/min/mm^2^), this effect was significantly lower compared to the fluid secretion observed in WT ducts (283 ± 67 pl/min/mm^2^, *P* = 0.0029; see Fig. 5).

### 8-Bromo cAMP

**Figure 6.**
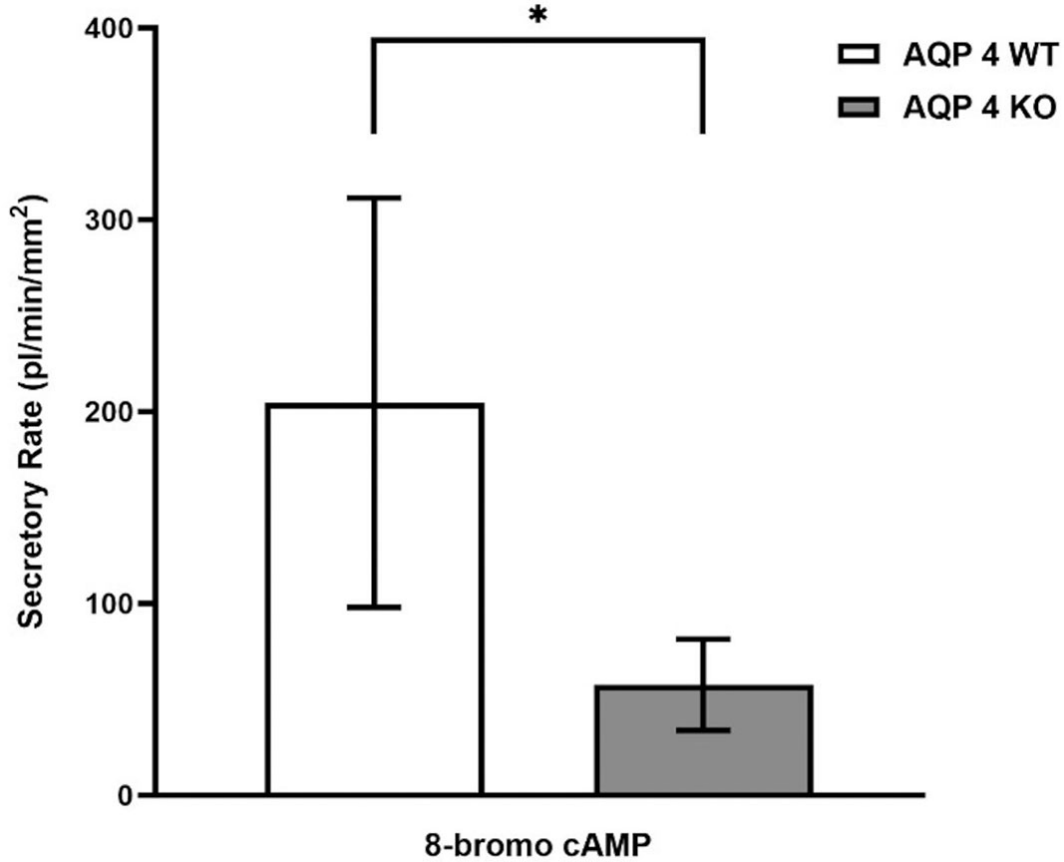
Effect of 100 µM 8-bromo cAMP on fluid secretion in mouse ducts isolated from WT and AQP4 KO mouse LGs. WT and AQP4 KO mouse ducts were exposed to 100 µM 8-bromo cAMP. Secretory rates are shown. Data were obtained from seven ducts isolated from four different animals and are presented as means ± SEM. Fluid secretory capability was significantly reduced in AQP4 KO mouse ducts.

Elevation of intracellular cAMP levels proved to be a potent stimulator of fluid secretion in our previous studies in both rabbit and mouse LG ducts.^7^^,^^9^ To investigate whether AQP4 is involved in the secretory effect of adenylyl cyclase/cAMP pathway, isolated ducts were treated with the cell-permeable cAMP analogue 8-bromo cAMP. Although 8-bromo cAMP (100 µM) induced significant fluid production in both WT and AQP4 KO mouse ducts (WT = 219 ± 92 pl/min/mm^2^ and AQP4 KO = 58 ± 23 pl/min/mm^2^), the effect of 8-bromo cAMP was significantly higher in WT ducts (*P* = 0.0015; Fig. 6).

### Phenylephrine

**Figure 7.**
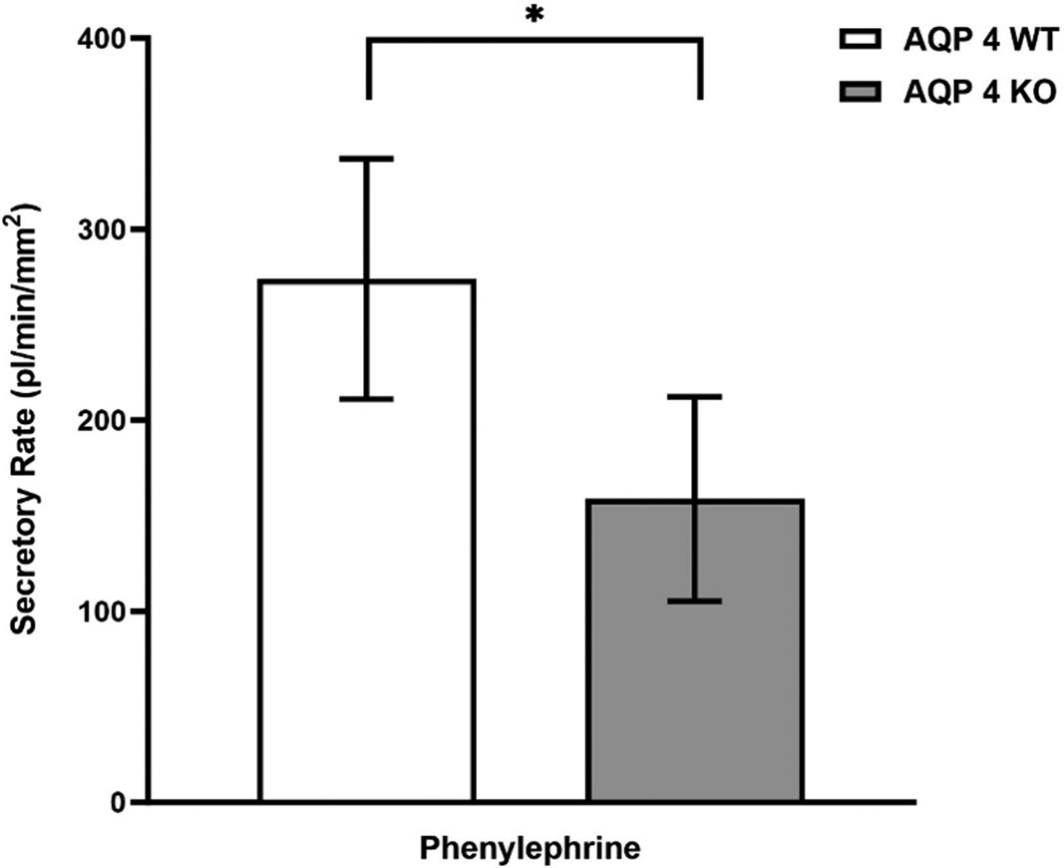
Effect of 10 µM phenylephrine on fluid secretion in mouse ducts isolated from WT and AQP4 KO LGs. WT and AQP4 KO ducts were exposed to 10 µM phenylephrine. Secretory rates are shown. Data were obtained from six ducts isolated from four different animals and are presented as means ± SEM. Secretory rate was significantly lower in ducts isolated from AQP4 KO animals.

Besides the apparent role of parasympathetic agonists, a substantial outcome of α-adrenergic activation on LG ductal secretion was also proven in our previous experiments.^10^ To assess the role of AQP4 in the α-adrenergic stimulation-induced fluid secretion, PHE (10 µM) was applied. Although PHE induced a relevant secretory response in both WT and KO ducts, this response was significantly lower in ducts isolated from AQP4 KO LGs (WT = 274 ± 63 pl/min/mm^2^ and AQP4 KO = 158 ± 53 pl/min/mm^2^, respectively, *P* = 0.006; Fig. 7).

## Discussion

AQP transmembrane channels are crucial elements of transcellular water transport in exocrine glands, however, their distribution and functional relevance vary depending on tissue and species.^12^^,^^13^ In the case of the LG, the expression of AQP1, 2, 3, 4, 5, 7, 8, 9, and 11 have been confirmed in different species so far, but their exact roles have yet to be clarified.^14^^–^^16^

Although the purpose of the present study was to explore the role of AQP4 in ductal fluid secretion, we additionally measured the overall impact of AQP4 in tear fluid production. In these experiments, the global amount of tear produced by the lacrimal functional unit (LFU) of WT and AQP4 KO mice did not differ, providing evidence that the presence of AQP4 is not required for the maintenance of basal tear secretion. However, these results do not reflect the involvement of AQP4 in the function of different parts of the LFU.

In our further experiments, we investigated the secretory properties of duct epithelia from WT and AQP4 KO mouse LGs using our isolated LG duct model and video-microscopy. P_f_ is a calculated parameter characteristic of the water permeability of the tissue. In our experiments, no significant difference in P_f_ could be observed between WT and AQP4 KO mouse duct epithelium. The lack of AQP4 protein, therefore, does not seem to modify the basic characteristics of ductal transepithelial water permeability.

Understanding of the regulation of AQP4 remains a challenging area. AQP4 seems to be regulated through different signaling ways involving potential transcriptional and translational alterations, as well as direct binding of ligands.^26^ Recent studies have shed light on different aspects of AQP4 regulation. The activation of AQPs via cGMP or cAMP has been extensively investigated. Hidehiro et al. found that in rat optic nerve astrocyte cell culture an elevated intracellular NO level causes an elevation in the expression of AQP 4 via the cGMP/protein kinase G pathway.^27^ In another study by Baetz and colleagues, an increase in the amount of fluid transport was observed in retinal pigment epithelial cultures after the administration of a cell-permeable cGMP analogue.^28^ Studies have shown that VIP has a regulatory effect on AQPs, which may involve the cAMP/PKA signaling pathway.^29^^,^^30^ Upregulated cAMP/PKA signaling can result in the phosphorylation/activation of various AQP channels, including AQP3, AQP4, and AQP8.^31^^,^^32^ However, further investigation is needed to address many unresolved questions in this area.

To clarify the regulatory machinery of AQP 4 in LG duct cells, ductal fluid secretion was investigated under stimulatory conditions. Various types of secretagogues were administered to the bath solution in both WT and AQP4 KO mouse ducts, and the ductal secretion was observed. The secretory response evoked by elevated cytosolic cAMP levels (via cell permeable 8-bromo-cAMP and VIP) and sympathetic stimulation proved to be significantly lower in AQP4 KO mouse ducts compared to WT, whereas cholinergic stimulation-related responses were similar. Immunofluorescence staining of VIP receptors (VPAC1 and VPAC2) resulted in a similar staining pattern and density in both WT and AQP4 KO mouse ducts, indicating that the significantly greater ductal tear secretion induced by VIP in WT ducts is not related to altered receptor expression.

In order to contextualize our results within the physiological function of the LG duct system, it is necessary to understand the basics of the autonomic regulation of the lacrimal ducts. Physiologically, the autonomic regulation of LG secretion is controlled by both the parasympathetic and sympathetic nervous system. It has long been established that parasympathetic pathway is the dominant route in the regulation of LG function. The parasympathetic axon terminals can be classified into two branches based on the neurotransmitters secreted: cholinergic nerve endings release acetylcholine, whereas VIP is produced by the VIP-ergic nerve terminals. Both neurotransmitters have a significant impact on LG secretion.^33^^–^^35^ Cholinergic agonists elevate the intracellular Ca^2+^ level, whereas the main intracellular event of VIP-ergic stimulation is the increase of cytosolic cAMP level, and to a much lesser extent, the elevation of cytosolic Ca^2+^ levels. Earlier, we reported the secretory effect of the cholinergic agonist carbachol on isolated LG ducts in both rabbits and mice, as well as the direct fluid secretory effect of VIP-ergic stimulation on isolated LG duct segments from mice.^7^^,^^9^^,^^33^ Although the determining regulatory pathway of LG is the parasympathetic route, recent evidence suggests the relevant influence of the sympathetic nervous system through the α_1D_ receptor using the NO/cGMP (and elevated cytosolic Ca^2+^ level to a much smaller extent) intracellular pathway in the fluid secretion of LG duct epithelium in mice.^10^

As we found in our previous studies, besides the parasympathetic-cholinergic pathway, both the parasympathetic-VIP-ergic and sympathetic stimulation also play important roles in regulating the volume of the secreted fluid of LG ducts. In our present study, we found that these latter signaling pathways can be related to the presence of AQP4 channels, whereas the role of the calcium-dependent cholinergic pathway is not significant in this regard. These results may be consistent with the widely accepted notion and with our own findings that in the case of the LG AQP channels play certain, partly still unknown roles, but the absence of the AQP proteins does not necessarily influence basal tear secretion / LG function under physiological conditions.

AQP4 is predominantly expressed in the ductal cells of the LGs, with much weaker abundance in the acinar cells. The functional relevance of AQP4 in acinar cells, specifically its role in tear secretion through these cells in not well defined. The AQP channel-related water transport in LG acinar cells is attributed to AQP5, rather than AQP4. In summary, although AQP4 have some presence in the LG acinar cells, its significance and role in tear secretion from acinar cells are not as relevant as that of AQP5. The precise role of AQP4 in lacrimal gland acinar cell function and tear secretion requires further exploration. Future research may provide additional insights into how AQP4 contributes to LG physiology and ocular surface hydration.

Currently, most data are available on the role of AQP5. Numerous animal studies and human research data are available regarding the role of AQP5 in experimental autoimmune dacryoadenitis and Sjögren's syndrome. The decrease in AQP5 protein expression, along with defective transcellular transport and altered distribution, may be associated with the development of dry eye disease in these conditions. These findings suggest that AQP channels are suggested to have a significant impact on the pathophysiological processes of LG function.^36^ Further clarification of the role of AQP5 holds promise for the development of specific therapeutic interventions, such as gene transfer, aimed at restoring the function of the channel. These interventions could potentially restore lacrimal gland function in cases of dry eye disease.^37^

In conclusion, our present findings indicate that parasympathetic-VIP-ergic and sympathetic stimulations require the active involvement of AQP4 channels. In contrast, the parasympathetic-cholinergic stimulatory pathway remains unaffected by the presence or absence of AQP4 channels. Likewise, basic tear secretion does not depend on the active contribution of AQP4 channels, and the water permeability characteristics of the ductal epithelial cells will also remain unchanged when AQP4 channels are absent.

Our results may shed light on the physiological processes underlying previously published controversial findings on the involvement of AQP4 in LG secretion. Furthermore, our present findings regarding AQP4 function in ductal secretion strengthen the notion that the duct system in the LG plays important role in the modification of the composition of the primary acinar fluid and therefore can have a decisive role in finalizing the secretion produced by the LG. Further research on the specific roles of AQP water channels in LG physiology and pathophysiology will enhance our understanding of tear film dynamics and may serve as potential therapeutic targets for ocular surface disorders.
